# hMT+ activity predicts the effect of spatial attention on surround suppression

**DOI:** 10.1167/jov.25.4.12

**Published:** 2025-04-23

**Authors:** Merve Kınıklıoğlu, Huseyin Boyaci

**Affiliations:** 1 Department of Neuroscience, Bilkent University, Ankara, Türkiye; 2 Aysel Sabuncu Brain Research Center & National Magnetic Resonance Research Center (UMRAM), Bilkent University, Ankara, Türkiye; 3 Department of Psychology, Bilkent University, Ankara, Türkiye; 4 Department of Psychology, Justus-Liebig University Gießen, Gießen, Germany; 5 Present address: Mathematical Institute, Justus-Liebig-University Gießen, Gießen, Germany

**Keywords:** spatial attention, surround suppression, response normalization, motion perception, middle temporal cortex

## Abstract

Surround suppression refers to the decrease in behavioral sensitivity and neural response to a central stimulus due to the presence of surrounding stimuli. Several aspects of surround suppression in human motion perception have been studied in detail, including its atypicality in some clinical populations. However, how the extent of spatial attention affects the strength of surround suppression has not been systematically studied before. To address this question, we presented human participants with “center” and “surround” drifting gratings and sought to find whether attending only to the center (“narrow attention”) versus both to the center and surround (“wide attention”) modulates the suppression strength in motion processing. Using psychophysics and functional magnetic resonance imaging (fMRI), we measured motion direction discrimination thresholds and cortical activity in the primary visual cortex (V1) and middle temporal complex (hMT+). We found increased perceptual thresholds and, thus, stronger surround suppression under the wide-attention condition. We also found that the pattern of hMT+ activity was consistent with the behavioral results. Furthermore, a mathematical model that combines spatial attention and divisive normalization was able to explain the pattern in the behavioral and fMRI results. These findings provide a deeper understanding of how attention affects center–surround interactions and suggest possible neural mechanisms with relevance to both basic and clinical vision science.

## Introduction

Sensitivity to a visual stimulus strongly depends on its surround. Notably, discriminating the motion direction of a drifting central grating becomes harder when presented with iso-oriented high-contrast surround (Tadin, Lappin, Gilroy, & Blake, 2003). This perceptual surround suppression phenomenon is frequently attributed to the antagonistic center–surround interactions in the visual cortex, particularly in the middle temporal cortex (Er, Pamir, & Boyaci, 2020; Pack, Hunter, & Born, 2005; Schallmo et al., 2018; Tadin et al., 2003; Tadin, Silvanto, Pascual-Leone, & Battelli, 2011; Turkozer, Pamir, & Boyaci, 2016). Stimulus characteristics, such as size and contrast, have been shown to influence neural (Pack et al., 2005; Pihlaja, Henriksson, James, & Vanni, 2008; Schallmo et al., 2018; Turkozer et al., 2016; Williams, Singh, & Smith, 2003; Zenger-Landolt & Heeger, 2003) and behavioral suppression (Er et al., 2020; Schallmo et al., 2018; Tadin et al., 2011; Turkozer et al., 2016). However, previous research on the effect of attention on surround suppression in human motion processing is relatively limited.

Previous animal studies have shown neural correlates of the attention affecting surround suppression in general, including in motion processing (Herrmann, Montaser-Kouhsari, Carrasco, & Heeger, 2010; Ito & Gilbert, 1999; Flevaris & Murray, 2015a, 2015b; Freeman, Sagi, & Driver, 2001; Maunsell, 2015; Reynolds & Chelazzi, 2004; Sundberg, Mitchell, & Reynolds, 2009). Human neuroimaging studies, on the other hand, have demonstrated the effect of attention on surround suppression across a variety of features except for motion (Flevaris & Murray, 2015a; Itthipuripat, Garcia, Rungratsameetaweemana, Sprague, & Serences, 2014; Schallmo, Grant, Burton, & Olman, 2016). In a recent behavioral study, we examined the effect of the spatial extent of attention on surround suppression in human motion perception using drifting gratings presented in different size and contrast levels and found that increasing the spatial extent of attention causes stronger surround suppression (Kınıklıoğlu & Boyaci, 2022). So far, however, there has been no human neuroimaging study examining how the spatial extent of attention modulates the neural correlates of surround suppression in human motion perception.

To fill this gap, we studied the effect of attention on surround suppression using behavioral and functional magnetic resonance imaging (fMRI) experiments. For this purpose, we measured motion direction discrimination thresholds and fMRI responses in human primary visual cortex (V1) and middle temporal complex (hMT+) using drifting high-contrast central gratings presented together with large annular gratings under two attention conditions: a narrow-attention condition, in which participants were instructed to attend only to the center grating, and a wide-attention condition, in which they were asked to attend to both the center and surround. To anticipate, consistent with our previous study (Kınıklıoğlu & Boyaci, 2022), we found stronger perceptual suppression under the wide-attention condition compared to the narrow-attention condition. Next, we examined whether the strength of neural suppression in V1 and hMT+ reflected this behavioral effect. Finally, we used a mathematical model, namely the normalization model of attention (NMA) (Reynolds & Heeger, 2009), to explain the behavioral and fMRI data and, thus, establish a link between them.

## Methods

### Participants

Ten volunteers (mean age = 25.7 years, eight female) participated in both the behavioral and fMRI experiments of the study. All participants reported normal or corrected-to-normal vision and had no history of neurological or visual disorders. Participants gave their written informed consent before the experiment. The experimental protocols were approved by the Human Ethics Committee of Bilkent University.

### Behavioral experiment

#### Apparatus

The visual stimuli were presented on a mid-gray background (16.09 cd/m^2^) using a CRT monitor (HP P1230, 22 inches, 1,280 × 1,024 resolution, 120 Hz refresh rate) in a dark room. Participants viewed the stimuli at a distance of 75 cm, with their heads stabilized by a chin rest. Stimuli were programmed with the Psychophysics Toolbox (Brainard, 1997) on MATLAB 2018b (MathWorks, Natick, MA, USA). A gray-scale look-up table was prepared through direct measurements of the luminance values (SpectroCAL, Cambridge Research Systems Ltd., Cambridge, UK) and used to ensure the presentation of correct luminance values.

#### Stimuli and design

**Figure 1.**
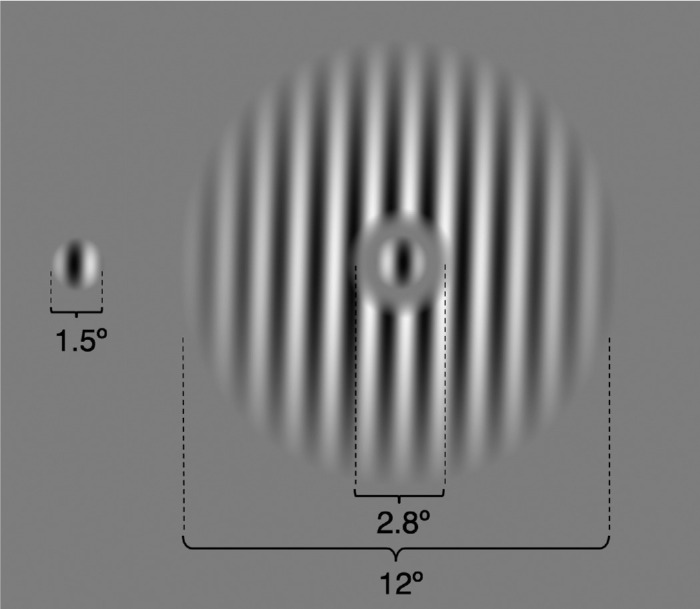
Drifting gratings in center-only and center + surround configurations.

The stimuli, shown in Figure 1, matched those described previously (Kınıklıoğlu & Boyaci, 2022). Briefly, we used drifting sinusoidal gratings (frequency: 1 cycle/degree, speed: 4°/s, starting phase randomized) weighted by two-dimensional raised cosine envelopes, the radius of which defined the stimulus size. The stimulus consisted of a center grating surrounded by an annular grating (except for the center-only configuration; see below). The Michelson contrast of both center and surround gratings was 98%.

In both the narrow-attention and wide-attention conditions, the center grating was presented with a surround grating. The center-only trials were used as a baseline to calculate the suppressive effect of the surround on the center. The diameter of the center grating was 1.5°. The inner and the outer diameter of the surround were 2.8° and 12°, respectively. The area between them (1.5° to 2.8°) remained unstimulated to separate the center grating from the surround grating. The center and surround gratings drifted either in the same or opposite direction. For each trial, their directions were determined pseudorandomly such that, in half of the trials, they drifted in the same direction.

Narrow- and wide-attention conditions were tested in separate blocks, whereas the two direction conditions (same and opposite direction) were tested in the same block in randomized order. Center-only trials were tested in the narrow-attention blocks. The order of the blocks was counterbalanced for each participant and administered on the same day.

**Figure 2.**
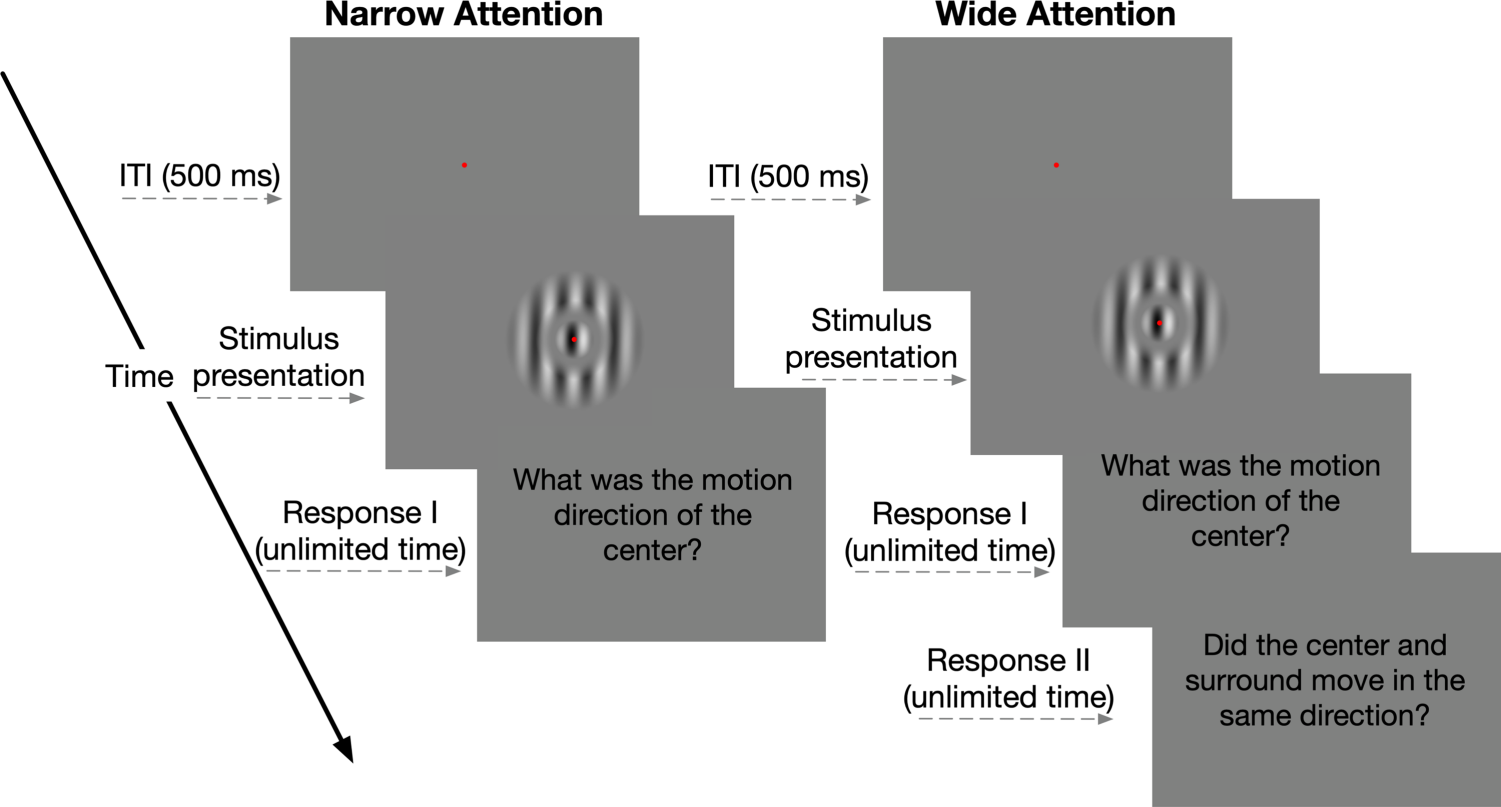
Trial sequence for the narrow- and wide-attention conditions in the behavioral experiment. Participants were asked to fixate on the center of the display throughout the trial. Each trial began with a fixation point, followed by the presentation of the stimulus, the duration of which was adjusted with two interleaved 1-up, 3-down staircases. In the narrow-attention condition, participants reported the drift direction of the center grating. In the wide-attention condition, participants first reported the drift direction of the center grating and then reported whether the center and surround gratings drifted in the same direction.

Throughout the experiment, participants viewed foveally presented drifting gratings while maintaining fixation at the center of the stimulus and performed motion direction discrimination tasks via a standard keyboard press. In the narrow-attention condition, participants were instructed to attend only to the center grating and report its motion direction. In the wide-attention condition, participants were asked to attend both the center and surround gratings. They first reported the drift direction of the center grating, then reported whether the center and surround gratings drifted in the same direction (Figure 2). This second question was solely used to encourage the participants to extend their spatial attention.

In each trial, the duration of the presentation, defined as two standard deviations (*SD*) of a temporal Gaussian envelope (Borghuis, Tadin, Lankheet, Lappin, & van de Grind, 2019; Tadin et al., 2003), was adjusted with two interleaved 1-up, 3-down adaptive staircases, based on the participant’s responses in previous trials. There were two independently progressing staircases for each condition. One staircase started from a very short duration (33 ms), which made the task relatively harder, and the other started from a long duration (158 ms), which made the task relatively easier. There were 100 trials in each staircase. Each participant completed 200 trials for center-only, 400 trials for narrow-attention, and 400 trials for wide-attention conditions. The experimental session took approximately 45 minutes. Brief break periods were given during the experiment.

#### Data analysis

Duration thresholds (79% success rate) were estimated by fitting a Weibull function to the proportion of correct responses using the Palamedes toolbox (Kingdom & Prins, 2010) in MATLAB 2019a (MathWorks) for each participant and condition. Next, using the threshold values, a suppression index, SI, was calculated to quantify the strength of the surround suppression

(1) $$\begin{array}{c} SI=\frac{T_{C+S}-T_{C}}{T_{C}},\; \end{array}$$

where *T*_*C*_ and *T*_*C* + *S*_ are the discrimination thresholds for center-only and center + surround configurations, respectively. Higher positive values of SI indicate stronger surround suppression, whereas negative SI values mean surround facilitation. An SI of 0 means no suppression or facilitation. Further statistical tests were performed on the SI values.

First, we compared the SI values to “0” by applying a one-sample, two-tailed Student's *t* test with correction for multiple tests using SPSS Version 25 (SPSS, Inc., Chicago, IL, USA). Next, we performed two-way repeated-measures ANOVA with factors attention (narrow and wide attention) and direction (same and opposite direction). Then, we conducted two post hoc paired sample *t* tests to further explore how the spatial extent of attention affects surround suppression in different direction conditions.

### fMRI experiment

#### Data acquisition

Magnetic resonance (MR) data were collected on a 3 Tesla Siemens Trio MR scanner (Magnetom Trio, Siemens AG, Erlangen, Germany) with a 32-channel head coil in the National Magnetic Resonance Research Center (UMRAM), Bilkent University. Anatomical data were acquired using a T1-weighted three-dimensional (3D) anatomical sequence (TR: 2,600 ms, spatial resolution: 1 mm^3^ isotropic). Blood oxygen level-dependent (BOLD) functional images were acquired with a T2*-weighted echo-planar imaging (EPI) sequence (TR: 2,000 ms, TE: 35 ms, spatial resolution: 3 × 3 × 3 mm^3^). The stimuli were presented on a 32-inch (1,360 × 768, 60 Hz) MR-compatible LED monitor (T-32; Troyka Med A.S., Ankara, Turkey). The monitor was placed near the rear end of the scanner bore and viewed by the participants from a distance of 156 cm via a mirror attached to the head coil. The stimuli were generated and presented using MATLAB and the Psychophysics Toolbox (Brainard, 1997). Participants’ responses were collected via an MR-compatible fiber-optic response box (Current Designs, Philadelphia, USA). The session started with an anatomical scan, followed by three localizer and six experimental functional runs, and took approximately 1 hour in total.

#### Experimental runs

**Figure 3.**
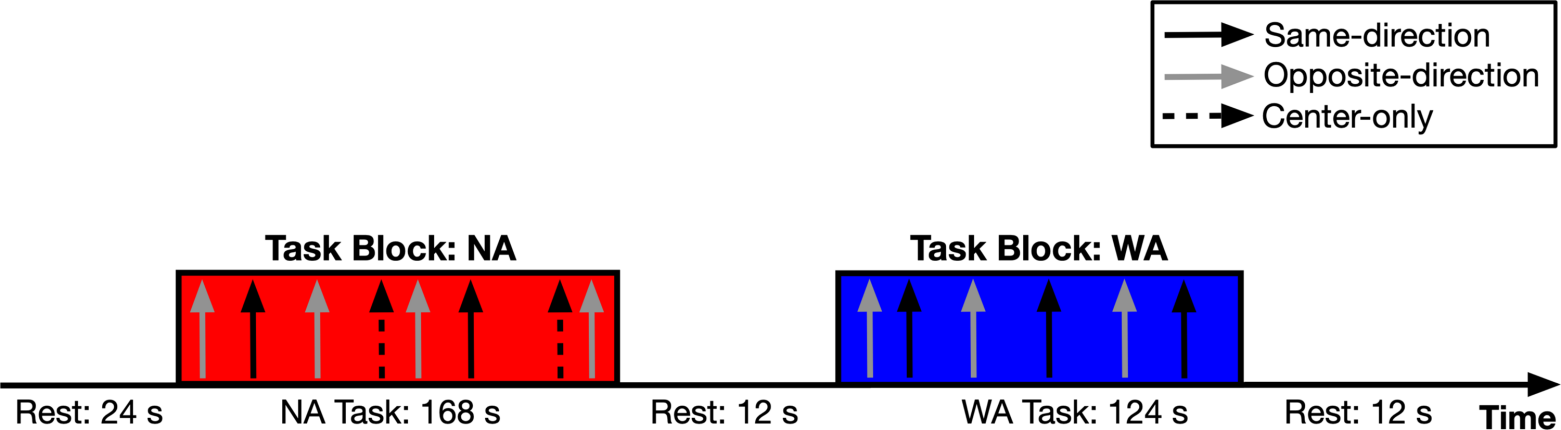
Schematic depiction of the mixed design of the fMRI experiment. In each run, task blocks were alternated with rest blocks. The order of two block types (NA: narrow attention; WA: wide attention) was randomly determined. The narrow-attention block consisted of center-only, same-direction, and opposite-direction trials. Wide-attention blocks consisted of same- and opposite-direction trials. Within a task block, each condition was repeated six times and the intertrial intervals were jittered between 4 and 8 seconds.

The stimuli were drifting sinusoidal gratings as in the behavioral experiment (Figure 1). We used a mixed design in which narrow-attention blocks alternated with wide attention blocks. The center-only trials were presented in the narrow-attention blocks. The experimental design is depicted in Figure 3. Two conditions of attention (narrow and wide attention) were tested in separate blocks, whereas two conditions of direction (same and opposite direction) were tested in the same block in a randomized order. Each run started with a 24-s rest, followed by a 168-s narrow-attention and a 124-s wide-attention task block in a randomized order, interleaved by a 12-s rest block, and ended with a 12-s rest. Task blocks started with an instruction screen. Within a task block, each condition (three conditions for narrow attention and two conditions for wide attention) was presented six times, and the interval between trials (ITI) was jittered between 4 and 8 seconds. The total duration of a functional run was around 5 minutes. There were six experimental runs in the session.

**Figure 4.**
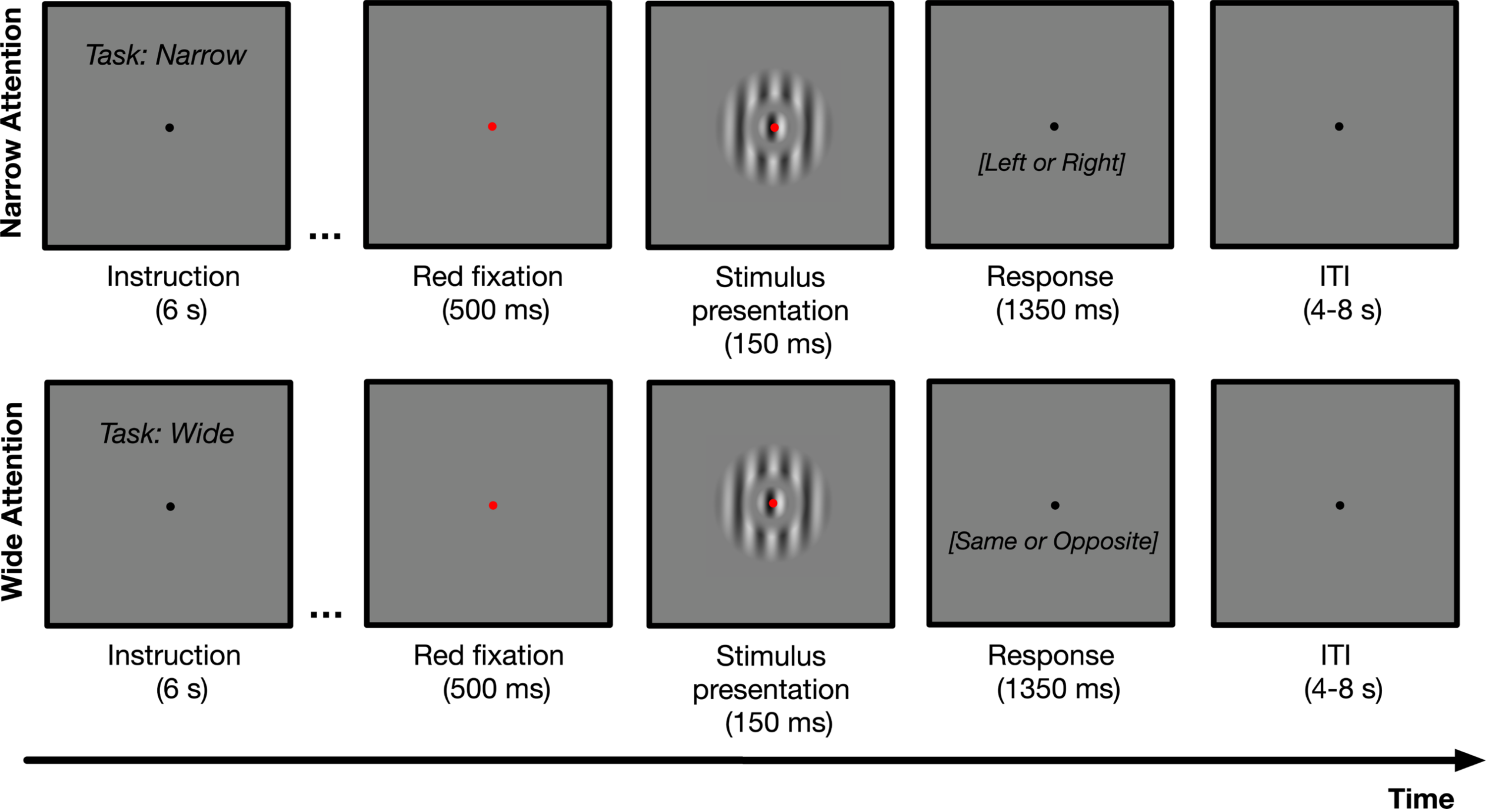
Trial sequence of the narrow- and wide-attention blocks in a functional run. Participants were asked to fixate on the center of the display throughout the run. Each block started with instructions about the task. In the narrow-attention block, the participants were instructed to attend only to the center grating and report its drift direction. In the wide-attention block, the participants were instructed to attend both center and surround gratings and report whether the center and surround gratings drifted in the same or opposite directions. Each trial began with a fixation point, followed by the presentation of the stimulus for 150 ms. In the experiment, possible answers were not written on the screen during the response period.

Stimuli were presented for 150 ms on a mid-gray background. As in the behavioral experiment, the participants viewed foveally presented drifting gratings and performed a task on the perceived drift direction via an MR-compatible response box. In the narrow-attention and center-only conditions, participants were asked to attend to the center grating and report its drift direction. In the wide-attention condition, the participants were instructed to attend both to the center and surround gratings and report whether the center and surround gratings drifted in the same direction (Figure 4). Unlike the behavioral experiment, we asked only one question in the wide-attention condition since asking two consecutive questions could elicit confounding neural activity.

#### hMT+ and V1 localizer runs

To localize participants’ hMT+ areas, we collected fMRI data in a separate run using standard methods in the literature (Huk, Dougherty, & Heeger, 2002). Specifically, we used a field of moving and static dots, which consisted of 100 randomly positioned white dots presented foveally within a 12-degree diameter circular aperture on a black background. The dots moved in three different trajectories: cardinal (left–right; up-down), angular (clockwise–counterclockwise), and radial (expanding–contracting). The direction of motion was altered every 2-s to prevent adaptation. The run started with a 24-s blank period, followed by 12-s blocks of a field of static dots alternating with 12-s blocks of a field of dynamic dots. There were six dynamic and static blocks in a run. Throughout the entire run, participants were required to maintain central fixation and perform a demanding fixation task in which they were asked to detect changes in the color of the fixation point.

To localize participants’ V1 areas, we collected fMRI data in a separate run. Similar to established retinotopic mapping methods in the literature (Engel, Glover, & Wandell, 1997; Sereno et al., 1995), we used flickering checkerboard-patterned wedges. However, as we were only interested in the V1/V2 boundary, instead of rotating and expanding wedges, we used alternating horizontal and vertical wedge stimuli (Greenberg et al., 2012; Slotnick & Yantis, 2003). During this localizer run, the presentation of 16-s horizontal wedge stimuli blocks alternated with 16-s vertical wedge stimuli blocks. This cycle was repeated eight times in a run. Throughout the entire run, participants were required to maintain central fixation and perform a demanding fixation task in which they were asked to detect changes in the color of the fixation point.

#### hMT+ and V1 sub-region of interest (ROI) localizer run

Using an independent localizer run, we identified the set of voxels corresponding to the spatial location and size of the center grating as sub-ROIs within hMT+ and V1. In this localizer run, participants viewed drifting high-contrast (98%) center-only and surround-only gratings whose size and location were the same as in the behavioral experiment and the functional runs (Figure 1). The run consisted of 12-s active blocks alternated with 12-s rest blocks, repeated six times. Center gratings and surround gratings were presented in different active blocks. Throughout the entire run, participants were required to maintain central fixation and perform a demanding fixation task in which they were asked to detect changes in the color of the fixation point.

### Data analysis

#### Preprocessing

MR data were preprocessed and analyzed using the FMRIB Software Library (FSL) (www.fmrib.ox.ac.uk/fsl) and FreeSurfer (Dale, Fischl, & Sereno, 1999; Fischl, Sereno, & Dale, 1999; Woolrich, Ripley, Brady, & Smith, 2001). High-resolution anatomical images were skull-stripped with BET. Preprocessing steps for functional images included motion correction with MCFLIRT, high-pass temporal filtering (100 s), and BET brain extraction. Each participant’s functional images were aligned to their own high-resolution anatomical image and registered to the standard Montreal Neurological Institute (MNI) 2-mm brain using FLIRT. Then, the 3D cortical surface was constructed from anatomical images for each participant using FreeSurfer’s *recon-all* command for visualizing statistical maps, anatomical delineation, and identifying ROIs.

#### ROI construction

For all ROI constructions, a general linear model (GLM) was applied using FSL’s FMRI Expert Analysis Tool (FEAT). Temporal autocorrelations were removed by applying FILM prewhitening (Woolrich et al., 2001).

For the hMT+ ROI, the statistical parametric maps (SPMs) of the dynamic versus static contrast (α threshold = 0.05, corrected) were registered to FreeSurfer and overlaid on the surface in the native space using the *tksurfer* command. Utilizing the MT label from FreeSurfer’s anatomical delineation for guidance, voxels at the ascending tip of the inferior temporal sulcus and responding more to dynamic compared to static dots were identified as hMT+ and used as a mask for the hMT+ sub-ROI localization.

For the V1 ROI, SPMs of horizontal versus vertical contrast (α threshold = 0.05, corrected) were registered to FreeSurfer and overlaid on the surface in the native space using the *tksurfer* program. Utilizing the V1 label from FreeSurfer’s anatomical delineation for guidance and voxels that respond stronger to vertical than horizontal wedges, we drew the V1–V2 boundaries. The voxels that fell in or around the calcarine sulcus were identified as V1 and used as a mask for the V1 sub-ROI localization.

For V1 and hMT+ sub-ROIs, we analyzed the independent localizer run data and identified the voxels that respond more strongly to the center compared to the surround grating within V1 and hMT+. The activated regions were identified as V1 and hMT+ sub-ROIs, respectively, and used for further analyses.

#### Analysis of experimental runs

Analysis of the experimental functional runs involved extracting GLM beta weights from sub-ROIs using FSL (Jenkinson, Beckmann, Behrens, Woolrich, & Smith, 2012; Smith et al., 2004). At the first level, we conducted event-related voxelwise analyses, where the predicted fMRI response in each trial was computed assuming a double-gamma hemodynamic response function (HRF). Nuisance regressors for linear motion (derived from MCFLIRT) were also included in the model. For removing the temporal autocorrelations, FILM prewhitening was applied (Woolrich et al., 2001). Contrasts were calculated, focusing on the main effect of each condition. The second level of the functional analysis involved a fixed-effects combination of all six runs for each participant, providing a per-participant average of the first-level contrasts.

Then, to compute the fMRI response within the hMT+ and V1 sub-ROIs, we extracted parameter estimates for each condition using the Featquery tool (FMRIB, Oxford, UK). Specifically, we extracted the beta weights for each trial type across all voxels within each of the predefined ROIs. We treated these extracted beta weights as the fMRI response and performed further statistical analyses on them.

Next, to quantify the changes in fMRI response caused by the inclusion of surround grating and also to draw a link between behavioral and fMRI results, we calculated an SI, defined as

(2) $$\begin{array}{ccc} & & SI=B_{C}-B_{C+S};\;C=center,\\ & & \;C+S=center+surround,\; \end{array}$$

where *B*_._ is the fMRI response for the given condition. A negative SI means surround facilitation, and a positive SI means surround suppression. An SI of 0 represents no suppression. Further statistical tests were performed on the SI values.

First, we compared the SI values to “0” by applying a one-sample, two-tailed Student’s *t* test with correction for multiple tests using SPSS Version 25 (SPSS, Inc.). Next, we performed a two-way ANOVA on the SIs with two factors: attention (narrow attention and wide attention) and direction (same and opposite direction) for each sub-ROI. Then, we conducted two post hoc paired sample *t* tests to further explore how attention affects surround suppression under different direction conditions.

### Model

To explain the behavioral and fMRI results, we used the NMA (Reynolds & Heeger, 2009), which incorporates attention into the computation of neurons’ responses. The model computes population responses as

(3) $$R\left(x,\theta ,c\right)=\frac{E\left(x,\theta ,c\right)\times M\left(x,\theta ,M_{g}\right)}{S(x,\theta ,c)+\sigma },$$

where *x* is spatial position; θ is drift direction; *c* is contrast; *E* and *S* are the excitatory and suppressive drives, respectively; and σ is a semi-saturation constant. The excitatory drive is

(4) $$E\left(x,\theta ,c\right)=e\left(x_{e},\theta_{e}\right)*N\left(x,\theta ,c\right),$$

where *e* is a two-dimensional (2D) Gaussian function that determines spatial and direction tuning, * denotes convolution, and *N* is a 2D Gaussian representing the stimulus. The suppressive drive is

(5) $$\;S\left(x,\theta ,c\right)=s\left(x_{s},\theta_{s}\right)*\left(E\left(x,\theta ,c\right)\times M\left(x,\theta ,M_{g}\right)\right),$$

where *s* is a broader 2D Gaussian tuning function compared to *e*, and *M* is a 2D Gaussian function whose width is set by the attentional gain factor *M*_*g*_. Smaller and larger *M*_*g*_ define narrow- and wide-attention fields, respectively. The operator “×” represents element-wise multiplication (Hadamard product). Opposite-direction trials are modeled by using surround stimulus direction that is 180° away from the direction of the center (Kınıklıoğlu & Boyaci, 2022; also see Reynolds & Heeger, 2009). Figure 8 pictorially summarizes the NMA components.

**Figure 5.**
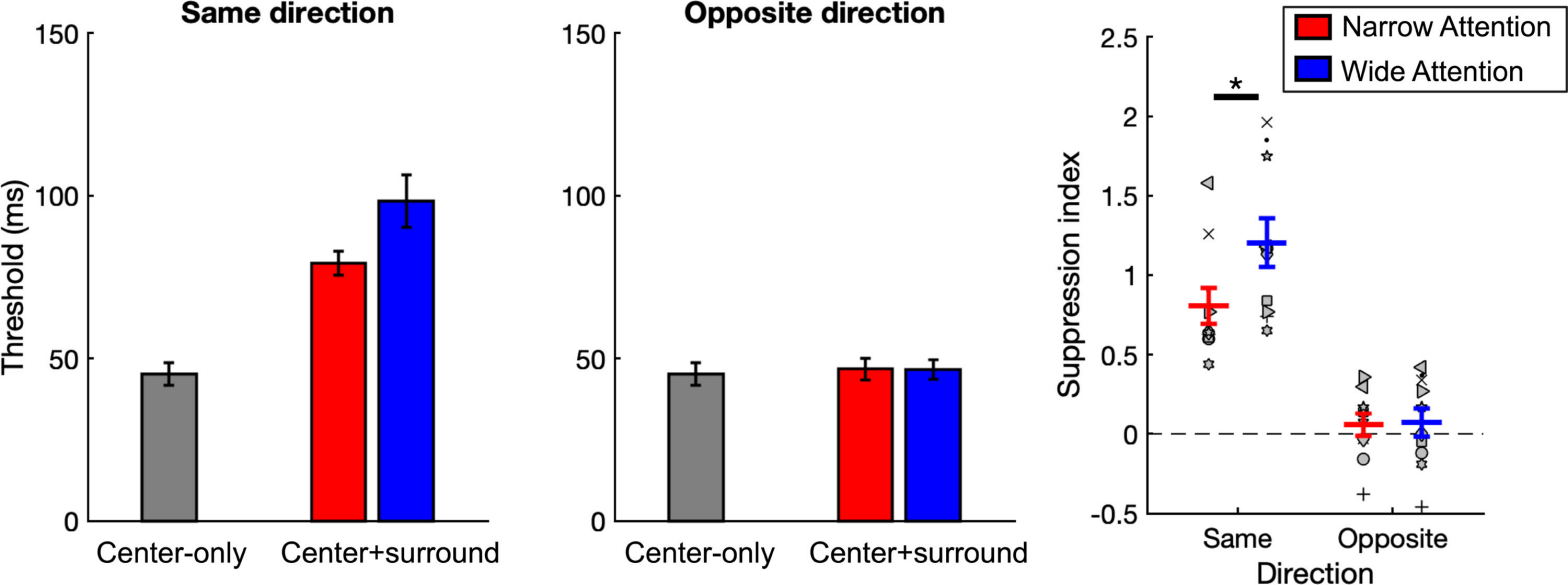
Behavioral results. Left two plots: Drift direction discrimination thresholds averaged across participants. Right plot: Suppression Indices (SI). Higher values of *SI* indicate stronger surround suppression, and negative *SI* values indicate surround facilitation. Symbols represent individual participants, and the red and blue lines show averages for narrow and wide attention conditions, respectively. *Indicates significance at *p* < 0.05. Error bars represent SEM.

**Figure 6.**
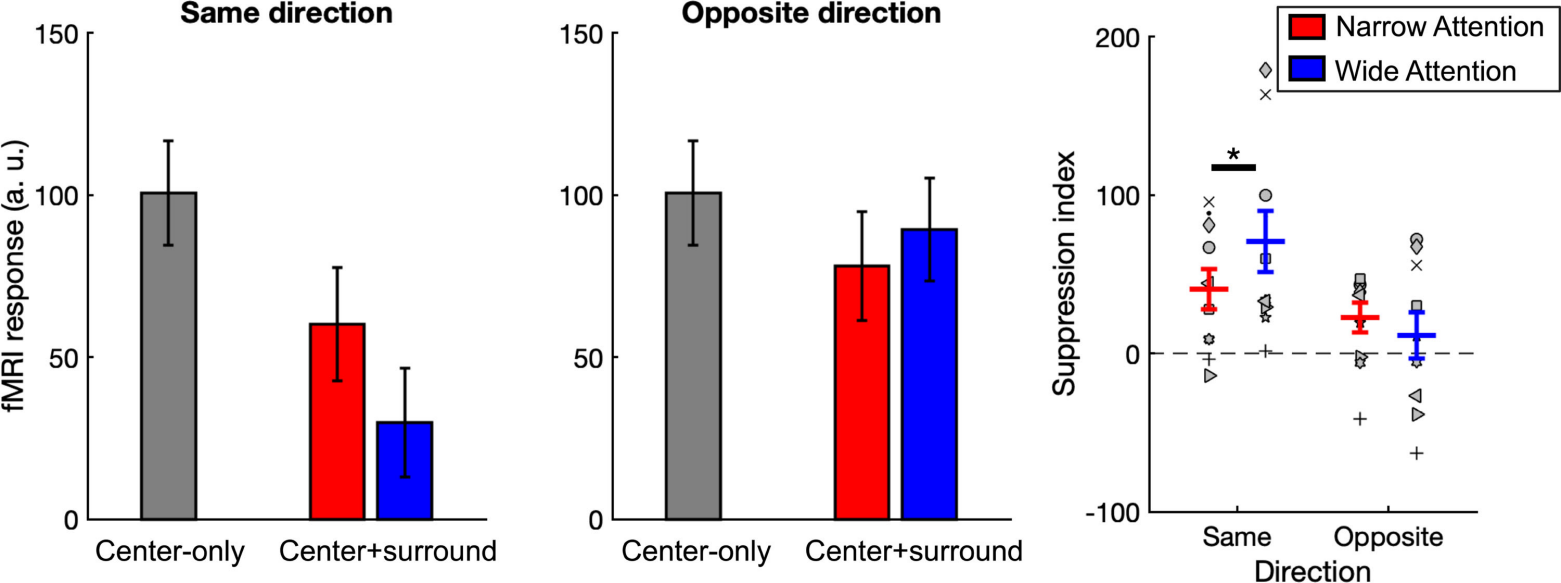
fMRI responses from hMT+ sub-ROI. Left two plots: fMRI responses averaged across participants. Right plot: Suppression indices (*SI*) derived from fMRI responses. Red and blue lines indicate narrow- and wide-attention conditions, respectively. *Indicates significance at *p* < 0.05. Error bars represent SEM.

**Figure 7.**
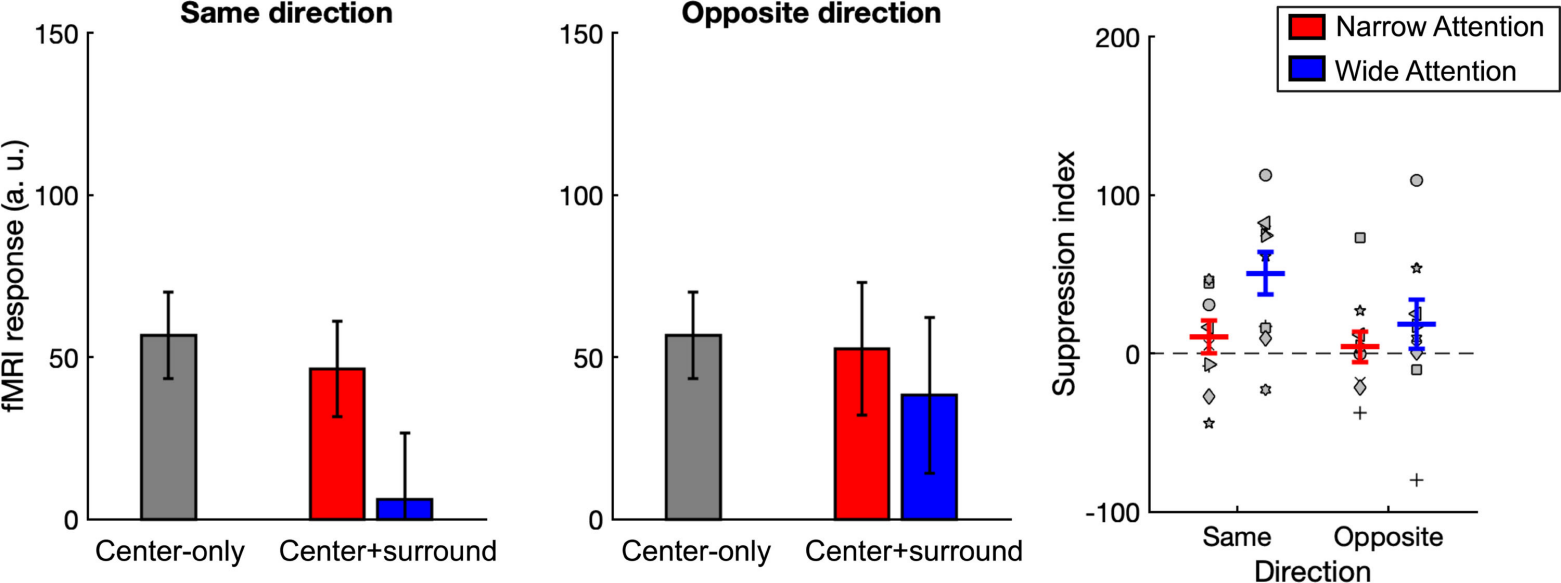
fMRI responses from V1 sub-ROI. Left two plots: fMRI responses averaged across participants. Right plot: Suppression indices (*SI*) derived from fMRI responses. Red and blue lines indicate narrow- and wide-attention conditions, respectively. Error bars represent SEM.

**Figure 8.**
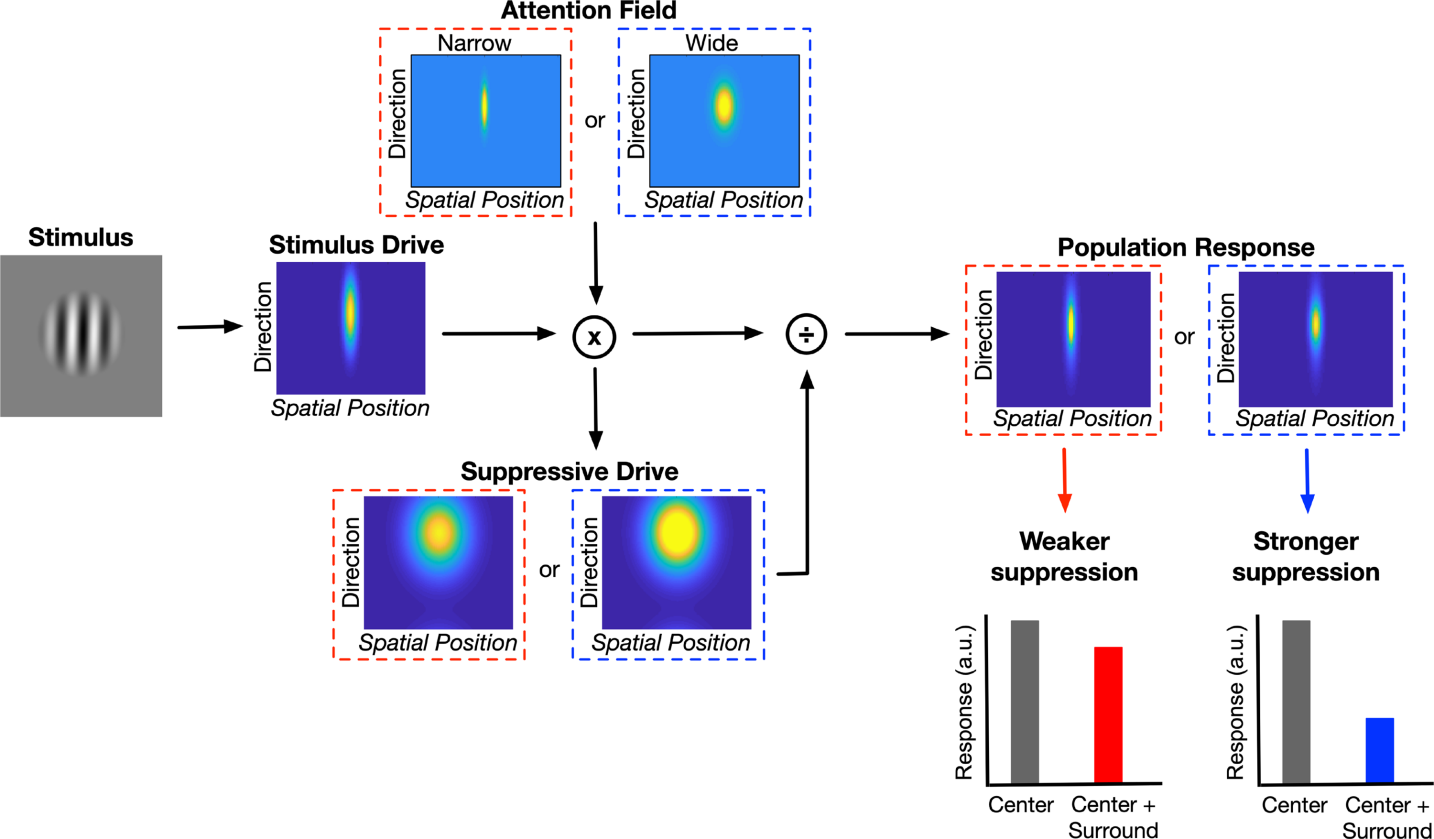
A schematic representation of the normalization model of attention. Stimulus drive is multiplied by the attention field, then normalized by the suppressive drive to determine population response. A narrow-attention field leads to a weaker suppression (red dashed squares), and a wider-attention field leads to a stronger suppression (blue dashed squares) (Equations 3–5).

Once the population responses were computed, we used the mean of the maximum responding population as a proxy for the fMRI response, which was calculated by

(6) $$\begin{array}{ccc} R_{peak} & \;= & mean(R(x_{max}-r_{w}:x_{max}+r_{w},\\ & & \theta_{max}\;-\;r_{w}:\theta_{max}+r_{w},c)), \end{array}$$

where *x*_*max*_ and θ_*max*_ are the values where *R* attains its maximum value, and *r*_*w*_ defines the width of the averaging area around this peak. Predicted motion discrimination thresholds were then computed as

(7) $$\begin{array}{c} T=\frac{C}{R_{peak}},\; \end{array}$$

where *C* is a constant linking the behavioral thresholds and neuronal responses.

The model was fitted to the fMRI data averaged across participants. Specifically, the average beta weights for all four conditions were normalized by the average beta weights of the center-only condition. The model was then fitted to the normalized fMRI data using five free parameters: excitatory spatial pooling width (*x*_*e*_), suppressive spatial pooling width (*x*_*s*_), excitatory direction pooling width (θ_*e*_), suppressive direction pooling width (θ_*s*_), and attentional gain factor (*M*_*g*_). Model parameters were estimated by minimizing mean squared errors (MSEs) averaged across conditions. This optimization was performed using custom functions (Er et al., 2020; Schallmo et al., 2020) and MATLAB’s *fmincon* function (version 2022b).

To validate the robustness of modeling results, a null distribution was generated by shuffling the beta weights 1,000 times, estimating the model parameters for each shuffled dataset and calculating the MSE values. To evaluate the model’s performance, fivefold cross-validation was conducted. In each iteration, 80% of the data was used as the training set, and the remaining 20% served as the test set. This process was repeated until each subset was used as the test set once. The model’s performance was assessed by calculating the MSE on the held-out test sets and averaging it across five iterations.

## Results

### Behavioral results

Figure 5 shows drift direction discrimination thresholds and the suppression indices (*SI*) derived from them. One-sample *t*tests showed that under both attention conditions, the *SI* values were significantly larger than zero in the same-direction trials (*p*s < 0.001) but not in the opposite-direction trials (*p*s > 0.05). Moreover, two-way repeated-measures ANOVA results revealed a significant main effect of attention (*F*(1, 9) = 5.48, *p* < 0.05, η^2^ = 0.38), a significant main effect of direction (*F*(1, 9) = 104.56, *p* < 0.001, η^2^ = 0.92), and a significant attention–direction interaction (*F*(1, 9) = 8.17, *p* < 0.05, η^2^ = 0.48).

Post hoc tests revealed stronger surround suppression under the wide-compared to narrow-attention condition in the same-direction trials, (paired-sample *t*test: *t*(9) = −2.75, *p* = 0.022; *a*_*corr*_ = 0.025), but not in the opposite-direction trials (*t*(9) = −0.24, *p* > 0.05).

### fMRI results

Figure 6 shows fMRI responses from hMT+ sub-ROI and suppression indices (*SI*) derived from them. One-sample *t*tests showed that under both attention conditions, the *SI* values were significantly larger than zero in same-direction trials (*p*s < 0.0125), not in opposite-direction trials (*p*s > 0.05). Moreover, two-way repeated-measures ANOVA revealed no main effect of attention (*F*(1, 9) = 1.05, *p* > 0.05, η^2^ = 0.10), but there was a significant main effect of direction (*F*(1, 9) = 19.89, *p* < 0.05, η^2^ = 0.69), as well as a significant interaction between attention and direction (*F*(1, 9) = 32.81, *p* < 0.001, η^2^ = 0.79).

Post hoc paired-sample *t*test results showed that *SI* values were significantly larger in the wide-attention condition compared to the narrow-attention condition only in same-direction trials (*t*(9) = 2.90, *p* = 0.018; *a*_*corr*_ = 0.025), but not in opposite-direction trials (*t*(9) = −1.18, *p* > 0.05), reflecting the pattern observed in the behavioral experiment.

Figure 7 shows fMRI responses from V1 sub-ROI and suppression indices derived from them. One-sample *t*tests revealed that *SI* values were significantly larger than zero only for wide-attention same-direction trials (*p* < 0.01; α_*corr*_ = 0.0125). For the opposite-direction trials and same-direction narrow-attention trials, *SI* values did not significantly differ from zero (*p*s > 0.05). Two-way repeated-measures ANOVA results showed a marginally significant effect of direction (*F*(1, 9) = 4.92, *p* < 0.054, η^2^ = 0.35). However, neither the main effect of attention nor the attention–direction interaction reached significance (*p*s > 0.05).

Post hoc paired-sample *t*test results showed that *SI* values were marginally larger in the wide-attention condition compared to the narrow-attention condition in same-direction trials (*t*(9) = 2.34, *p* = 0.044; *a*_*corr*_ = 0.025), and there was no difference between them in opposite-direction trials (*t*(9) = 0.88, *p* > 0.05)

Assuming a linear relationship between the fMRI response and neural activity (Boynton, Demb, Glover, & Heeger, 1999) and further assuming that behavioral thresholds decrease monotonically with increased neural activity, our results indicate that hMT+ responses reflect the behavioral effects. However, V1 activity is not consistent with the behavioral data, as we did not find an effect of attention in the same-direction trials, and there was no significant suppression in the narrow-attention condition. Consequently, our modeling approach in the following section will focus on the hMT+ and behavioral data.

### Model results

Here, we tested whether we could explain the hMT+ and behavioral responses using the NMA and whether the optimized parameter values are consistent with those from previous human studies based on monkey research. Our implementation of NMA is summarized in Figure 8, and details are described in the Methods section. Briefly, we first compute neural population responses using the divisive normalization model by incorporating a two-dimensional attention field for space and direction. Next, assuming a linear relation between them, we compute the fMRI responses from these neural responses. Finally, we compute the behavioral thresholds from the fMRI responses, assuming an inverse relation between the two.

**Figure 9.**
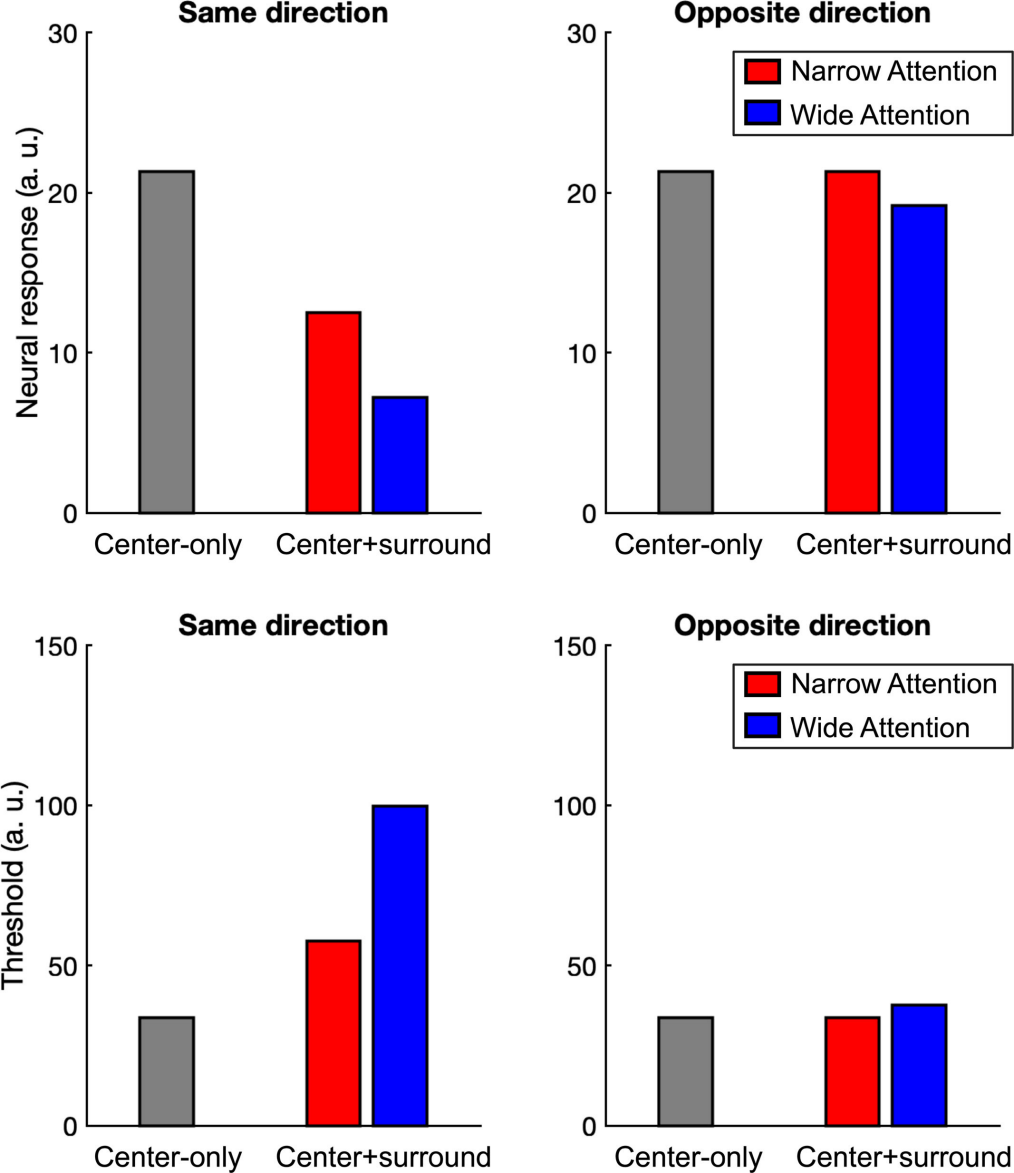
Model results. Top row: Model fit to hMT+ data. Bottom row: Model’s predictions of the behavioral results.

The model was fitted to the normalized fMRI data using five free parameters, which were estimated by minimizing MSE averaged across conditions. Table 1 shows the optimized parameter values, and Figure 9 shows the model predictions for fMRI and behavioral data.

**Table 1.** Normalization model parameters with their standard error of the mean (SEM) values in parentheses. The last two columns show the optimized parameter values from this study (SEM computed using fivefold cross-validation) and the values estimated from previous monkey neurophysiology studies (SEM computed across studies), respectively.

Symbol	Description	Optimized	Literature
*x*_*e*_	Excitatory spatial pooling width (a.u.)	3.68 (0.48)	4.5 (0.29)
*x*_*s*_	Suppressive spatial pooling width (a.u.)	40.67 (0.12)	36.25 (3.75)
θ_*e*_	Excitatory direction pooling width (°)	24.02 (0.31)	23.75 (1.25)
θ_*s*_	Suppressive direction pooling width (°)	49.34 (0.22)	47.5 (2.5)
*M*_*g*_	Spatial width of attention field—narrow (a.u.)	3.04 (0.26)	–
*M*_*g*_	Spatial width of attention field—wide (a.u.)	36.47 (4.32)	–

The model successfully fits the hMT+ data as indicated by the low MSE values for the observed data (MSE = 0.01). Additionally, the model qualitatively captures behavioral results (Figure 9). Furthermore, consistent with the task, the optimized value of *M*_*g*_ is smaller for the narrow-attention and larger for the wide-attention conditions (Table 1). Critically, the optimized model parameters closely align with those reported in previous human studies, which were based on parameter estimates derived from monkey research, as shown in the last column of Table 1. Notably, the values listed in this column represent the average of the parameters derived from several similar studies in the literature (Er et al., 2020; Kınıklıoğlu & Boyaci, 2022; Schallmo et al., 2018; Schallmo et al., 2020).

To validate the robustness of our modeling results, we generated a null distribution by shuffling the beta weights 1,000 times, estimating the model parameters for each shuffled dataset, and calculating the MSE values. The MSE for the model fitting of the observed data was not significantly different from the MSEs in the null distribution (observed MSE = 0.01; null mean MSE = 0.02; *p* > 0.05), but the estimated parameters deviated substantially from those derived from the original data and failed to align with previously reported estimates (Er et al., 2020; Kınıklıoğlu & Boyaci, 2022; Schallmo et al., 2018; Schallmo et al., 2020). Notably, the estimated attention field width (*M*_*g*_) was greater for the narrow-attention condition compared to the wide-attention condition, opposite to the patterns observed in the original fMRI data. Constraining the parameter estimates to ensure a wider attention field for the wide-attention condition resulted in a significantly higher MSE for the shuffled data compared to the original data (original = 0.01; shuffled = 0.07; *p* < 0.05), confirming that the model's fit was not due to chance.

Model performance was further evaluated using fivefold cross-validation, with 80% of the data for training and 20% for testing in each iteration. The average MSE across all folds was 0.04 (±0.01), indicating that the model is stable and performs consistently across different subsets of the data.

## Discussion

Here, we replicated our previous behavioral findings (Kınıklıoğlu & Boyaci, 2022) and showed that the strength of perceptual surround suppression increases as the extent of spatial attention increases. Specifically, when the center and surround gratings drift in the same direction, temporal direction discrimination thresholds are larger with wider spatial attention compared to narrower spatial attention. There is, however, no effect when the gratings drift in opposite directions. Our fMRI results showed that hMT+ activity is consistent with this pattern: When the center and surround gratings drift in the same direction, fMRI responses to the center grating are smaller with wider spatial attention compared to narrower spatial attention, and there is no effect when the gratings drift in opposite directions. Finally, performing numerical simulations, we show that a mathematical model, namely the divisive normalization model of attention (Reynolds & Heeger, 2009), can explain the hMT+ and behavioral responses, thus providing a link between behavioral and cortical responses.

To the best of our knowledge, these results provide the first neural evidence for the effect of the extent of spatial attention on surround suppression in human motion processing. The general effect of the spatial extent of attention on neural activity in the visual cortex was previously studied (Itthipuripat et al., 2014; Liu et al., 2022). For example, Itthipuripat et al. (2014), studying steady-state visual evoked potentials (SSVEPs), showed that focused attention enhances the neural signal-to-noise ratio compared to distributed attention. Similarly, a larger neural response in human V1 is observed when attention is directed to the static center grating compared to surrounding flanker gratings. In motion processing, previous animal studies have frequently reported attentional modulation in MT (Anton-Erxleben, Stephan, & Treue, 2009; Büchel et al., 1998; Lee & Maunsell, 2009; Lee & Maunsell, 2010; Ni, Ray, & Maunsell, 2012; Stoppel et al., 2011; Treue & Maunsell, 1996). Notably, Anton-Erxleben et al. (2009) demonstrated that in macaque MT, the strength of surround suppression increases for the attended location compared to the unattended location by increasing the influence of the attended stimulus on the center neuron’s firing rate. Our findings align well with those earlier results and show that similar neural mechanisms may underlie the effect of attention on human motion processing.

Surround suppression is reduced or eliminated when the center and surround move in opposite directions. This has been shown in both neurophysiology studies (Allman, Miezin, & McGuinness, 1985; Born & Tootell, 1992; Cavanaugh, Bair, & Movshon, 2002; Kastner, Nothdurft, & Pigarev, 1995; Lamme, 1995) and behavioral studies (Paffen, Alais, & Verstraten, 2005; Paffen, van der Smagt, te Pas, & Verstraten, 2005). Consistent with those studies, under the opposite-direction condition, we did not observe surround suppression, and we did not find an effect of the extent of spatial attention on it in our behavioral and fMRI results. Note that this also ensured that our results were not due to a task-demand artifact. There are, on the other hand, several human fMRI studies reporting that the opposite direction of surround motion facilitates cortical activity for the center (Takemura, Ashida, Amano, Kitaoka, & Murakami, 2012; Moutsiana, Field, & Harris, 2011). In those studies, however, authors investigated the effect of motion direction using motion aftereffect (MAE) and induced motion paradigms, not surround suppression, which might explain the difference in the results.

Divisive normalization and its variant incorporating attention (NMA; Reynolds & Heeger, 2009) have been successfully used to model the correlates of surround suppression in humans using model parameter values derived from monkey studies (Er et al., 2020; Kınıklıoğlu & Boyaci, 2022; Schallmo et al., 2018; Schallmo et al., 2020). However, the validity of the model using parameter values obtained directly from human neural data has not been shown before. We found that NMA predicts human fMRI and behavioral data simultaneously, and, importantly, the optimized parameter values closely match those reported in previous human studies based on monkey research. These results demonstrate that divisive normalization and NMA are valid tools to model the correlates of surround suppression in humans and that they can establish a link between the perceptual effects and neural responses.

We found that a model incorporating multiplicative attention gain can explain the surround suppression effects observed in the current study. In contrast, previous studies suggested that attention gain could be additive (Poltoratski, Ling, McCormack, & Tong, 2017; Schallmo et al., 2016), potentially due to differences in stimulus configuration and experimental design. For example, in these studies, targets and contextual elements were positioned across a broad area of the visual field with different attention manipulations. Poltoratski et al. (2017) used spatial cues to shift attention between closely spaced targets in opposite hemifields, likely resulting in a more restricted attention field than in our design. Schallmo et al. (2016) examined fMRI responses during tasks involving attention directed to specific targets compared to fixation. In our study, the target and surround were positioned closely, with attention allocated either to the target alone or to both the target and surround. These differences likely account for the contrasting findings.

The pattern of activity in V1 does not completely agree with the behavioral results. This could be related to overall surround suppression mechanisms in motion processing. According to the MT-hypothesis, surround suppression in motion processing originates in hMT+, not V1 (Tadin et al., 2003). Thus, V1 activity may not reflect any effect of attention on surround suppression. Our current results and a number of prior fMRI studies support the MT-hypothesis, showing that hMT+ activity mirrors the behavioral outcomes of surround suppression (e.g., Er et al., 2020; Schallmo et al., 2018; Tadin et al., 2011; Turkozer et al., 2016). Findings about V1 activity are mixed. Some studies found suppression in V1, suggesting that the surround suppression effect could be inherited from V1 by hMT+ (Angelucci, Bijanzadeh, Nurminen, Federer, Merlin, & Bressloff, 2017; Nurminen, Kilpeläinen, Laurinen, & Vanni, 2009; Nurminen, Kilpeläinen, & Vanni, 2013; Zenger-Landolt & Heeger, 2003), whereas several other studies reported both suppression and facilitation depending on presentation and attention conditions (Flevaris & Murray, 2015a; Williams et al., 2003). In the current study, because of several limitations, we cannot completely rule out V1’s potential role. One such limitation is related to the fMRI technique. Our voxel size was 3 × 3 × 3 mm^3^, which might be too large to distinguish the V1 sub-ROI activity in response to a small center grating, leading to a reduced signal. Furthermore, because of the foveal confluence, the fMRI response from the V1 sub-ROI might have mixed the activity of several other early visual areas. Thus, although we did not find a good agreement between the behavioral and V1 findings, this may not mean the absence of V1’s role in the perceptual effect.

Weaker surround suppression is observed in multiple clinical conditions, including schizophrenia (Tadin et al., 2006), major depressive disorder (Golomb et al., 2009), and autism spectrum disorder (ASD) (Foss-Feig, Tadin, Schauder, & Cascio, 2013; Schallmo et al., 2020; Sysoeva, Galuta, Davletshina, Orekhova, & Stroganova, 2017). The reason underlying this phenomenon, however, is still not fully understood. Considering that attention mechanisms are affected in many clinical disorders (Clayton, Richards, & Edwards, 1999; Habermann-Paelecke, Pohl, & Leplow, 2005; Kreither et al., 2017; Luck & Gold, 2008), it is possible that attentional abnormalities lead to atypical visual processing in these patients. This idea is supported by a study that reports weaker surround suppression with an increased level of acetylcholine, which is a neuromodulator thought to be critically involved in attentional processing by leading to more focused voluntary spatial attention (Kosovicheva, Sheremata, Rokem, Landau, & Silver, 2012). Moreover, Schallmo et al. (2020) found weaker suppression in ASD patients, and their modeling work suggested that this could be due to narrower spatial attention, which is in line with our findings. Taken together, these results suggest that the reduced surround suppression found in clinical populations may be caused by abnormal attention mechanisms.
